# Preventing Falls in the Community Using an Integrated Healthcare and Community Provider Interprofessional Team

**DOI:** 10.1093/geroni/igaf122.2638

**Published:** 2025-12-31

**Authors:** Jennifer Drost, Susan Fosnight, Brandi Chrzanowski, Joseph Machiano, Michele Gareri, Kristin Baughman, Michelle Hughes, Margaret Sanders

**Affiliations:** Summa Health System, Akron, Ohio, United States; Summa Health System, Akron, Ohio, United States; Direction Home Akron Canton Area Agency on Aging & Disabilities, Green, Ohio, United States; Summa Health System, Akron, Ohio, United States; Summa Health System, Akron, Ohio, United States; Northeast Ohio Medical University, Rootstown, Ohio, United States; Direction Home Akron Canton Area Agency on Aging & Disabilities, Green, Ohio, United States; Northeast Ohio Medical University, Rootstown, Ohio, United States

## Abstract

One in four older adults fall each year resulting in significant avoidable morbidity and mortality. To address the complex interactions between fall risk factors, effective fall prevention efforts require collaboration between medical and community-based providers. Our health system and local Area Agency on Aging developed an interprofessional model called Care Management Interdisciplinary Team (CMIT). CMIT care managers (Social Workers and nurses) complete pre-team evaluations including a falls risk assessment. Cases are presented by CMs to CMIT which includes a geriatric physician and pharmacy specialists. Recommendations are implemented by the CM and consumer’s primary care physician. These include home modifications, therapies, medication changes, and other prevention interventions. We evaluated pre-post team fall outcomes reported by CMs January-December 2023 (n = 64) using Chi Square. CMs reported that: 33% who had falling issues at baseline had no falls at 3 months (p < 0.0001), 52% who had medication concerns at baseline had those concerns resolved at 3 months (p = 0.03), 45% who had safety issues at baseline had those issues resolved at 3 months (p < 0.0001), 36% who had ED/hospital utilization issues at baseline had no utilization at 3 months (p < 0.001). Interprofessional, interagency collaboration can decrease fall risk by addressing both medical/pharmacy and social determinants of health. Given that healthcare costs associated with falls is currently $50 billion nationally, this integrated interprofessional and interagency model of care is highly cost-effective.

